# Acute Cystitis in a Transfeminine Patient: Assessment and Treatment of Urinary Tract Symptoms

**DOI:** 10.1111/jmwh.13696

**Published:** 2024-10-25

**Authors:** Janell Senda, Julia C. Phillippi

**Affiliations:** ^1^ Vanderbilt University School of Nursing Nashville Tennessee

**Keywords:** anti‐infective agents, bacteriuria, cystitis, health services for transgender persons, pyuria, transgender persons, urinary, urinary tract, urinary tract infection

## Abstract

Management of acute cystitis in a transfeminine patient is discussed as an example of treatment of urinary tract infections (UTIs). The case is an introduction for clinicians who typically care for cisgender women and wish to expand the populations they serve to include care of gender‐diverse individuals. This is supportive of the 2021 American College of Nurse‐Midwives Position Statement on Health Care for Transgender and Gender Non‐Binary People. Possible differential diagnoses for urinary symptoms in transfeminine patients are discussed, as well as relevant history taking, examination skills, and treatment guidelines for acute cystitis of patients with penises along with discussion of basic care for transgender individuals seeking midwifery or primary care services.

## CASE REPORT

*E.J. (pronouns she, her) is a healthy 38‐year‐old transfeminine person (assigned male at birth) who presents to the clinic with dysuria, urinary frequency, and urgency for 3 days. She describes her pain as a constant moderate deep burning in her suprapubic area “like when you have a full bladder” and deep urethral “burning” with micturition. The symptoms began after she used tape to “tuck” her external genitals (penis and testicles) for a social event. Initially, E.J. attributed her symptoms to irritation from the tape. She has tried increasing fluid intake and over‐the‐counter cranberry tablets with no relief. E.J. reports no* *history of sexually transmitted infections (STIs) or urologic pathology or procedures, including circumcision or gender‐affirming genital surgeries. Results from a review* *of systems are negative for fever; chills; nausea; vomiting; constipation; diarrhea; abdominal, rectal, or back pain; genital lesions or rashes; gonadal pain; pain with ejaculation; pain or discharge from urethra; difficulty starting or stopping stream of urine; and noticeable blood in the urine or ejaculatory fluid*.

*E.J. states she has been mutually monogamous for 3 years with her partner, a cisgender female. They have both had HIV, gonorrhea, and chlamydia testing since the beginning of their monogamy, and E.J. has no concerns about STIs. She and her partner rarely participate in penetrative penile‐vaginal intercourse and never penetrative penile‐anal sex. She reports comfort with her current sex life and does not report any sexual dysfunction. She and her partner do not use condoms, and her partner uses an intrauterine device for contraception. E.J. confirms she feels safe in her relationship and has not recently been the victim of sexual violence*.

*Current medications include spironolactone 200 mg orally once daily, estradiol valerate 20 mg subcutaneous every 2 weeks, sertraline 50 mg orally once daily, and an over‐the‐counter daily oral multivitamin. She reports* *taking no additional medications or supplements and follows up regularly with a gender‐affirming endocrinologist and her primary care provider*.

*Of note, E.J. also reports working at a new location without access to gender‐neutral bathrooms, and she often holds her urine for an 8‐hour shift and limits water intake to avoid using the restroom*.

*On examination, vital signs are normal: temperature 37.1 °C (98.8* °*F* *), heart rate 89, respiratory rate 16, and oxygen saturation* *99%. E.J. appears mildly anxious with a pleasant and normal affect; heart with regular rate and rhythm; lungs clear to auscultation bilaterally; skin without lesions or rashes; and bowel sounds present in all 4 quadrants. Very mild suprapubic discomfort is noted on palpation, but other areas of the abdomen are nontender. There is no costovertebral angle tenderness. Consent was obtained for external genital examination. Penile tissue is without lesions or rashes; foreskin is unaltered . Urethral meatus is midline, and gonads are descended without erythema, tenderness, or masses. Following a shared decision‐making model, the midwife discussed with E.J. that although symptoms were suggestive of etiology other than prostatitis, it would be difficult to rule out this diagnosis without a digital rectal examination. E.J. then consented for this examination. Anus is intact without lesions or bleeding. Prostate is firm, fleshy, bilobed, approximately 2 cm in diameter, and nontender to palpation (normal findings)*.

*A point of care urinalysis was performed with pH of 6, specific gravity 1.015, and negative glucose, protein, bilirubin, urobilogen, and ketone results. Trace blood, moderate leukocytes, and positive nitrite results were noted. E.J. declined additional STI testing or bloodwork. Urine culture was sent; however, results were not available at this visit*.

*Based on the preliminary history, examination, and urinalysis, E.J. was diagnosed with acute cystitis and prescribed sulfamethoxazole‐trimethoprim (Bactrim) 800 mg/160 mg orally every 12 hours for 7 days. She was advised that treatment may be altered based on the urine culture results and antibiotic susceptibility. Other discharge instructions included to call or return to the office for signs of infection progression such as fever, vomiting, back pain, or symptoms such as gonadal pain or urethral discharge*.

*Tucking practices were also discussed, including taking regular breaks and using alternatives to tape such as tight‐fitting underwear or a gaff (compression underwear designed to minimize the appearance of a bulge in the crotch area.) The importance of hygiene, increased water intake, and voiding breaks was also reinforced. E.J. was referred to the National Center for Transgender Equality website for information on workplace rights and bathroom access. She was scheduled for a well visit and advised to continue current positive health behaviors. E.J. was notified 3 days later that her urine culture result was* *100,000 colony count of pan‐sensitive* Escherichia coli*. At that time, she stated all symptoms were resolved*.

*(This case is a composite of elements from different patients)*.

## BACKGROUND

Sexuality, anatomical sex, and gender are complex and nuanced concepts that deserve to be considered individually. However, from the perspective of the clinician, a patient's genital anatomy at the time of a visit is an important variable contributing to their risk for urinary disease processes, as well as a differentiator for treatment. As access to gender‐affirming care has expanded, the spectrum of anatomical variation a clinician may encounter has also expanded. Phrases like *transfeminine* or *assigned male at birth* do not provide sufficient information for treatment decisions. Therefore, throughout this case study, *patients with penises* or *penile tissue* is used to capture both cisgender men and transwomen without a history of any urologic surgery. As gender is a personal concept, many patients might not identify with this terminology and have other terms they wish to use for their external genitals. It is best to defer to the patient‐preferred terms.^1^ Norms surrounding these concepts are constantly evolving, and cisgender persons must look toward the trans community for guidance, which is subject to change over time.

Current guidelines on the assessment and diagnosis of urinary tract symptoms vary depending on the anatomy of the urinary tract. Urinary tract infections (UTIs), including acute cystitis, are less common in healthy adult people who have penises than in individuals with vaginas. These lower rates are thought to be related to structural differences, specifically urethral length. In either case, acute cystitis should be treated with antibiotics to reduce risk of complications.^2^ Treatment durations are often longer for those with penile tissue, although evidence suggests treatment length longer than 7 days is counterproductive.^2^, ^3^

When a transfeminine or other adult with a penis presents with a UTI, assessment includes comorbidities and behaviors that could increase the risk of infection. Risk factors for UTIs include intact foreskin, poor hygiene or voiding practices, insertive anal sex, uncontrolled HIV infection, history of urologic surgery or divergent anatomy, and tucking.^4^, ^5^, ^6^, ^7^, ^8^

There are a variety of possible differential diagnoses related to urinary tract symptoms in those with a penis and external gonads (see Table 1). Urinary tract infection, for example, refers to the presence of pathologic bacteria in the urinary tract in clinically relevant concentrations, typically greater than 10^5^ colony forming units per milliliter in a urine culture.^8^ Cystitis  refers to inflammation of the bladder.^7^, ^8^ This condition presents as dysuria, urinary frequency, urinary urgency, and sometimes hematuria.^8^ Cystitis can be acute or chronic (sometimes called interstitial) depending on the duration and pattern of symptoms. Acute cystitis is caused by infection and is further categorized as uncomplicated or complicated. Uncomplicated cystitis refers to mild to moderate disease occurring in healthy, nonpregnant individuals. Complicated cystitis can occur in those who are immunocompromised or other individuals at risk of developing severe disease.^8^

**Table 1:** Potential Diagnosis for Dysuria and Genitourinary Concerns in Patients With a Penis and External Gonads

Condition	Description	Differentiating Subjective Concerns	Differentiating Objective Findings
Acute cystitis	Cystitis is inflammation of the bladder.^7^ Acute cystitis is a disease process caused by infectious organisms ascending the urethra.	Dysuria, frequency, urgency.^7^	Possible suprapubic tenderness on palpation. Hematuria, pyuria, nitrituria on urinalysis possible.
Pyelonephritis	Severe type of UTI in which the primary disease burden is in the kidneys. This often occurs from an untreated or inadequately treated acute cystitis that ascends the urinary tract.^8^	Dysuria, body aches, lethargy, nausea/vomiting, flank pain, chills.^8^	Person is ill‐appearing, has a fever, or CVA tenderness is present. Hematuria, pyuria, nitrituria on urinalysis possible.
Renal Calculi	Formation of hard deposits in the kidney that become painful when they begin to travel down the ureter.	Dysuria, gross hematuria, flank pain.	CVA tenderness, hematuria without pyuria or nitrituria on urinalysis likely.
Epididymitis/ orchitis	Inflammation of the epididymis, a tube at the back of the testicle that transports sperm to the urethra.^9^, ^10^ Acute epididymitis is often caused by the ascending bacteria (either STI or UTI related) from the urethra.^9^, ^10^ Symptoms typically overlap with acute cystitis but can also include pain with ejaculation, blood in ejaculate, fever, chills, and a tender, swollen, or red testicle (orchitis).^9^ Orchitis can be from viral or bacterial causes, sometimes spread from the epididymis.^11^	Testicular pain, pain with ejaculation, blood in ejaculate.	Tenderness to epididymis and or tenderness to testicle(s), possible erythema and edema to scrotum. Hematuria possible on urinalysis.
Prostatitis	Inflammation of the prostate, can be classified as acute or chronic.^8^, ^11^ Acute prostatitis is most commonly caused by bacteria traveling from the urethra or bladder to the prostate and is sometimes a sequalae of untreated UTI or STIs.^11^	Dysuria, body aches, lethargy, back pain, nausea/vomiting, pelvic pain, rectal pain, pain in or behind testicles, pain with ejaculation, pain with bowel movement, blood in ejaculate, difficulty starting or stopping stream of urine.^8^, ^11^	Person often appears ill or has a fever, Prostate is boggy, enlarged, or tender on digital rectal examination. Hematuria, pyuria, or nitrituria on urinalysis possible.
Urethritis	Inflammation of the urethra, often caused by bacterial infection. Commonly from STIs including gonorrhea, chlamydia, and trichomoniasis.^8^	Dysuria, urethral discharge, rashes, history of new partners.^8^	Edema/erythema at urethral meatus, hematuria, pyuria, nitrituria on urinalysis possible.

Abbreviations: CVA, costovertebral angle, STI, sexually transmitted infection; UTI, urinary tract infection.

## PREVALENCE AND RISK FACTORS

Lifetime risk of UTI in people assigned male at birth who are uncircumcised is 32.1%, a 23% greater risk than those who are circumcised.^6^ It is believed that the foreskin, although protective against damage and irritation to the penile glans, can trap bacteria; therefore, circumcision can decrease the risk of UTI.^6^, ^12^ However, the incidence of UTI in healthy people assigned male at birth is low compared with those assigned female, and rates decrease sharply after infancy, likely due to cessation of diapering and glans maturation.^12^, ^13^ Good hygiene practices likely mitigate risk.

Most pathogens that cause acute cystitis originate from gastrointestinal bacteria spread to the urethra from the rectum.^8^ Although even impeccable hygiene cannot eliminate fecal bacteria, careful wiping following a bowel movement, routine washing of undergarments, and gentle cleansing of genital skin may reduce UTI risk.

Many types of sexual activity contribute to the migration of bacteria from the rectum. Being the insertive partner during anal sex increases the risk of bacteria entering the urethra. Condom use and voiding following penetrative sex, especially anal sex, can be helpful in risk reduction.^14^

Regular urination decreases infection by flushing bacteria from the bladder and urethra. However, poor voiding practices, such as holding urine for long periods, can contribute to UTIs as bacteria are allowed to multiply in the bladder. Anatomical variations, including congenital and surgical alterations, can disrupt routine flushing of the urinary system and further contribute to UTIs.^14^

Tucking refers to the practice of reducing or eliminating any visible bulge by securing external genitalia backwards toward the buttocks, with or without boosting the testes into the inguinal canals.^4^, ^5^, ^15^ Tucking increases UTI risk due to proximity of the urethra to the anus and difficulty voiding at regular intervals.^4^, ^5^, ^15^ The tucking method used can also affect UTI risk. Sometimes tucking is achieved with tape, which further limits the ability to void and can cause skin irritation.^4^, ^5^, ^15^ The use of a gaff may be preferred. Gaffs are tight‐fitting undergarments that hold genitalia without adhesives. However, gaffs are often made of synthetic, nonbreathable fabrics that can harbor bacteria if not cared for properly.^4^, ^5^, ^15^

## HISTORY TAKING

Assessment of urinary tract concerns requires a thorough history, physical examination, and interpretation of key laboratory findings. In assessing a transfeminine individual presenting with urinary symptoms, a thorough review of systems will include consideration for urologic pathology such as prostatitis, sexually transmitted urethritis, epididymitis pyelonephritis, and renal calculi^8^, ^9^, ^11^ (see Table 1).

An accurate assessment of genital and urinary anatomy is a key factor in identifying risks for infection.^1^, ^15^ This history includes assigned sex at birth, congenital abnormalities of the genitals, and any genital surgery, including gender‐affirming surgery. Following this, the health care provider can systematically discuss individual anatomy such as penis, testicles, vagina, uterus, or ovaries as part of an *organ inventory*. This is also an appropriate time to ask the patient what their preferred terms are for these organs and what language they feel most comfortable with the provider using. Further characterization of voiding practices including frequency and timing of voiding, strength of the urinary stream, and visual characteristics of urine, as well as voiding difficulties and barriers, is essential. Personal and genetic family history of urinary calculi, prostate pathology such as cancer or benign prostatic hyperplasia, and renal disease should be documented. Finally, screening should be performed for recent genital injuries or changes in care of the genital and urinary tract, including new detergents, underwear, or manipulation of the genitals including tucking or muffing (a sexual act in which a person's inguinal canals are penetrated).

A sexual history includes types of activity and information regarding number, sex, and gender of current partners, as well as concerns about STIs.^16^ It is important that providers do not make assumptions regarding sexual activity based on an individual's gender expression, and that clinicians educate themselves on common sexual practices in the transfeminine community. Examining sources written for transwomen by transwomen is essential. Bellweather’s text, while written for lay people, is considered a seminal work about sexuality in transfeminine bodies and could be a valuable resource for clinician education.^17^ It is important to note that transgender individuals are also more likely than cisgender individuals to have a past or recent history of sexual trauma, and so a trauma‐informed approach to sexual history and physical examination is essential.^15^

## PHYSICAL EXAMINATION

A shared decision‐making approach regarding physical examination components is important. A complete examination of external genitalia should be done in the presence of a trained chaperone if the patient is comfortable. Examination should include inspection of the external genitals as well as a digital rectal examination if symptoms suggest prostatitis and should include palpation of the gonads if epididymitis is suspected.^9^, ^11^ Exquisite tenderness of either of these regions suggests pathology other than cystitis. This examination can be incredibly traumatic if not approached with care.^1^, ^15^ If the patient is uncomfortable with genital examination and wishes to defer or decline, adequate information could be obtained through a detailed history and patient self‐examination. During a self‐examination, the person is asked to confirm the presence or absence of tenderness or masses when palpating their external genitalia and perform as much inspection as possible. Ideally the patient can be provided with adequate light and a hand mirror to identify erythema, rashes, or lesions. For most people, self‐palpation of their own prostate through digital rectal examination is not possible.

Just as with all adults, the external genitals should appear healthy. The genital area as a whole should be nontender without erythema, edema, or rashes, with no evidence of discharge from the urethral meatus. Mild suprapubic tenderness might be present, but significant abdominal pain or costovertebral tenderness should not be.

The digital rectal examination can be conducted in a variety of positions based on patient and provider comfort as well as patient functioning and body habitus. Lithotomy and dorsal recumbent are positions easily achieved by many patients. Side‐lying options such as sims or lateral decubitus might be preferable for patients with difficulty laying back. Other positions such as knee‐chest, or standing position with elbows on table, offer access and visualization of the rectum but can evoke a sexual connotation and may therefore be avoided in a trauma‐informed examination, unless adopted per patient preference.

Like all sensitive examinations, good patient‐provider communication is essential. Explaining procedure in detail, obtaining consent, and checking in frequently are all part of trauma‐informed care. When the patient is ready, the digital rectal examination is conducted by placing a single gloved, lubricated finger into the rectum, with the provider's palm facing in the same direction as the patient's anterior pelvis. The provider palpates rectal walls for masses and characteristics of the prostate. See Table 2 for potential examination findings.

**Table 2:** Prostate Examination Findings

Expected	Pathologic
Smooth, fleshy bilobed “bulb”	Asymmetry or nodules Spongy texture
Diameter of 1‐2 in	Enlarged
Located on anterior surface of rectum, ∼2 in inside rectum	Intrusion into rectum, enlargement
Nontender. Pressure might elicit urge to urinate	Significant tenderness with pressure

Source: McGowan.^11^

## LABORATORY TESTING

Acute cystitis might manifest on urinalysis as any combination of hematuria, pyuria, or nitrituria. Any of these findings in the presence of symptoms should prompt empiric treatment. Urine cultures are the gold standard for diagnosis and should be sent in the setting of urinary concerns.^8^

According to the Centers for Disease Control and Prevention (CDC), if a patient is sexually active and younger than 25 years of age, or has a new or nonmonogamous partner or multiple partners, then sending urine specimens or urethral swabs for gonorrhea, chlamydia, and trichomoniasis is appropriate.^10^, ^18^ Shared decision‐making is useful to determine the need for STI testing.

Acute prostatitis is a serious illness that can be a sequelae of urethritis or cystitis. Pyuria, nitrituria, and hematuria are common findings with prostatitis as well as cystitis. Prostatitis must be assessed via prostate examination and cannot be ruled out through urinalysis alone.^11^, ^18^ If the patient has convincing symptoms and the prostate is tender, enlarged, and boggy on examination, diagnosis of prostatitis can be made solely on these findings, without further laboratory tests.^11^, ^18^ To ensure resolution, referral to urology may be appropriate.

## TREATMENT

Due to lack of evidence, there is little consensus on treatment regimens for acute cystitis in cisgender men and even less for the trans community.^7^, ^15^ Generally, treatment of acute, uncomplicated cystitis is similar in all adult patients without a history of urologic surgery or comorbidities. Regimens include slightly longer antimicrobial therapy durations of 5 to 7 days in healthy patients with penile tissue without pyelonephritis, or 10 to 14 days if pyelonephritis is suspected.^2^, ^3^, ^7^ Acceptable antibiotic regimens in the absence of pyelonephritis include fluoroquinolones, sulfonamides, phosphorics, nitrofurans, and beta lactams.^7^, ^19^ However, phosphorics, nitrofurans, and certain beta lactams have poor perfusion of the prostate, so it is important to assess for prostatitis prior antibiotic selection.^20^ In this case, E.J. was treated with sulfamethoxazole‐trimethoprim 800 mg/160 mg orally every 12 hours for 7 days, a selection that was later supported by the culture results.

If gonorrhea or chlamydia is suspected, the clinician might consider presumptive treatment based on current CDC guidelines. However, it is important to obtain urine cultures, as these antibiotics are not first‐line treatments for acute cystitis.

For persistent or recurrent UTIs in a transfeminine patient, referral to a gender‐inclusive urologist is appropriate to assess for a structural or functional problems. For individuals with prior gender‐affirming genitourinary surgery, all UTIs should be treated as complicated with referral to a urologist.^15^

## CONCLUSION

In this case, E.J. received appropriate care, including respect for her identity and evidence‐based medical treatment, resolving her acute cystitis. This case is an exemplar for clinicians engaging with gender‐diverse individuals in the clinical setting.

Transgender individuals must overcome substantial barriers in the health care system. Discrimination, bias, and provider knowledge deficits can lead to subquality care.^1^, ^15^ The 2021 American College of Nurse‐Midwives Position Statement on Health Care for Transgender and Gender Non‐Binary People states that providing services to people across the gender spectrum is within the scope of practice for certified nurse‐midwives and certified midwives.^21^ However, assessment and treatment of the genital tract in transfeminine patients may be new skills for clinicians trained prior to the implementation of the 2020 Core Competencies in Basic Midwifery Practice.^21^, ^22^

Creation of an evidence‐based and person‐centered environment is essential in improving access to and receipt of appropriate health care for transgender individuals and improving health equity.^15^ Midwives have long welcomed diverse individuals into reproductive care and provided safe and personalized services, so they are well positioned to extend care to and act as an advocate for the trans community and other gender‐diverse individuals.

## CONFLICT OF INTEREST

The authors have no conflicts of interest to declare.
